# Evaluating Collaborative and Autonomous Agents in Data-Stream-Supported Coordination of Mobile Crowdsourcing

**DOI:** 10.3390/s23020614

**Published:** 2023-01-05

**Authors:** Ralf Bruns, Jeremias Dötterl, Jürgen Dunkel, Sascha Ossowski

**Affiliations:** 1 Computer Science Department, Hannover University of Applied Sciences and Arts, 30459 Hannover, Germany; 2 CETINIA, University Rey Juan Carlos, Móstoles, 28933 Madrid, Spain

**Keywords:** crowdsourcing, data stream learning, multiagent systems, collaborative coordination, market-based coordination

## Abstract

Mobile crowdsourcing refers to systems where the completion of tasks necessarily requires physical movement of crowdworkers in an on-demand workforce. Evidence suggests that in such systems, tasks often get assigned to crowdworkers who struggle to complete those tasks successfully, resulting in high failure rates and low service quality. A promising solution to ensure higher quality of service is to continuously adapt the assignment and respond to failure-causing events by transferring tasks to better-suited workers who use different routes or vehicles. However, implementing task transfers in mobile crowdsourcing is difficult because workers are autonomous and may reject transfer requests. Moreover, task outcomes are uncertain and need to be predicted. In this paper, we propose different mechanisms to achieve outcome prediction and task coordination in mobile crowdsourcing. First, we analyze different data stream learning approaches for the prediction of task outcomes. Second, based on the suggested prediction model, we propose and evaluate two different approaches for task coordination with different degrees of autonomy: an opportunistic approach for crowdshipping with collaborative, but non-autonomous workers, and a market-based model with autonomous workers for crowdsensing.

## 1. Introduction

Explicit crowdsourcing is a problem-solving paradigm in which tasks are outsourced to an on-demand workforce of private individuals. Mobile crowdsourcing is often considered as a specific case of explicit crowdsourcing, where the outsourced tasks are spatial in character, and interested individuals use their personal mobile devices to temporarily join the crowd, find nearby tasks, complete the tasks, and earn rewards [1,2,3]. Mobile crowdsourcing has been used for a wide range of purposes:

- *Crowdsensing* systems [4,5] recruit crowdworkers to gather sensor data at locations of interest. There are many applications of crowdsensing in the social sector, the environmental sector, and the infrastructure sector, among others. Examples include measurements of air quality [6] and noise pollution [7,8], monitoring of road traffic [9] and road surface conditions [10,11,12], as well as photo acquisition [13]. In these scenarios, data are obtained from many different locations and then used to analyze, maintain, and improve the monitored infrastructure or environment.
- *Crowdshipping* systems [14] tackle the problem of last-mile parcel delivery by a crowdsourcing approach, i.e., to service the delivery leg from the logistics company’s parcel hub to the recipients’ home. Interested individuals can join to act as couriers. The aim is to use spare capacities in private vehicles and piggyback on trips that are made by private citizens anyway. In exchange for monetary rewards, the interested individuals pick up parcels (or any other kind of freight) and bring them to their destinations, altering their original route only for minor detours [15].

The crowd has many advantages compared to permanent staff or fleets of professional drivers. The crowdworkers are spatially distributed and can solve many tasks by taking short detours from their original routes, which is fast, cost-efficient, and sustainable. Furthermore, the crowd is a scalable workforce that can be contracted on a task-by-task basis, which makes the crowd suitable for short-term projects that would be impractical or costly otherwise.

Unfortunately, under some conditions, mobile crowdsourcing fails to meet all expectations. Tasks are frequently assigned to crowdworkers who later face difficulties to complete these tasks successfully, resulting in high failure rates, unreliable system behavior, and low service quality. Task execution takes place in highly dynamic urban environments with many sources of disruption such as traffic and vehicle issues. From traditional delivery fleets, we know that unexpected events related to traffic, vehicles, and weather can result in large delays, high costs, and inferior customer service [16]. For instance, traffic jams can introduce huge delays to travel plans of workers moving by car. Weather conditions may put delivery deadlines at risk, especially if the means of transportation is a bicycle. Furthermore, during task execution, crowdworkers can be affected by unexpected situations, distraction, inexperience, tiredness, or competing obligations. For instance, fatigue can be a reason for a worker walking or cycling to deliver parcels later than expected [2]. Conventional crowdsourcing provides evidence indicating that tasks are frequently abandoned [17,18]. Such events can result in the delayed completion or failure of tasks. To ensure high service quality and long-term worker engagement, task failures must be prevented.

A possible method to prevent task failures, which has been successfully exploited in related domains [19,20], is to respond to failure-provoking events by strategically transferring tasks to more promising workers. If one worker is unwilling or unable to continue a task, another worker nearby may be willing and capable to complete the task instead. Task transfers are a promising technique because the crowd is heterogeneous. Crowdworkers use different transportation modes; they have different skills, experience levels, and preferences, and they have different destinations, routes, and travel patterns. Exploiting this heterogeneity, tasks that are executed too slowly by pedestrians can be executed more quickly by car drivers. On the other hand, bikes are less affected by road congestion and can travel under such conditions faster [21]. Hence, car drivers stuck in traffic may transfer their tasks to unaffected cyclists. Task transfers allow tasks to be executed faster and the system to behave more reliably. However, task transfers among crowdworkers have to deal with two main challenges:

- *Outcome uncertainty:* While a task is being performed by a crowdworker, the outcome of that task is uncertain, and it is generally unknown whether the task could be successful. However, the decision to transfer a task depends largely on the likelihood that the task can be completed successfully. Therefore, to enable task transfers, task outcomes must be accurately predicted.
- *Coordination:* Task transfers between workers must be coordinated. In general, the crowdworkers involved in a task transfer may have different levels of autonomy. Here, we distinguish between *collaborative workers*, who must perform a transfer proposed by the system in any case and *autonomous workers*, who can decide for themselves which task transfers will be carried out. Only if the task transfers are in line with the individual interests of the autonomous workers will they find acceptance and be executed by the workers. Even if transfers are efficient from the system’s point of view, autonomous workers will reject them if they are not beneficial to themselves.

In this paper we argue that supporting peer-to-peer task transfers among crowdworkers is a promising approach to prevent task failures in mobile crowdsourcing. In the following, we propose different mechanisms to achieve outcome prediction and task coordination. In particular, we set out from outcome prediction mechanisms outlined in [22] and compare a collaborative task coordination model to a market-based mechanism geared towards an open system of autonomous agents in line with [2].

More specifically, our main contributions are:

- To mitigate the outcome uncertainty, we propose a machine learning approach on data streams that can predict task outcomes.
- Based on the suggested prediction model, two different approaches to task coordination are proposed:
  (i) First, we present how non-autonomous collaborative workers cooperate by means of task transfers in the crowdshipping domain.
  (ii) We then propose a market-based model for autonomous workers using a crowdsensing application scenario as an example. In this approach, autonomous crowdworkers can perform or reject tasks according to their personal interests.

The paper is organized as follows. In Section 2, we discuss some related work. Then, Section 3 outlines how uncertainty can be tackled using continuously monitored sensor data and data stream learning. It presents a crowdshipping approach based on collaborative crowdworkers. In Section 4, we present how worker autonomy can be implemented in a crowdsensing scenario by coordinating task transfers through a market-based negotiation mechanism. Finally, we conclude the paper pointing to future lines of work in Section 5.

## 2. Related Work

So far, task transfers have been insufficiently addressed in the context of mobile crowdsourcing. Only for the crowdshipping domain do a few publications exist with the explicit aim to introduce task transfers. One of the earliest proposals in this direction was made by Sadilek et al. [14]. In their system, parcels can be transferred between workers if there is an overlap in space and time. They view mobile crowdsourcing with task transfers primarily as a routing and planning problem that can be solved with graph planning algorithms.

The assumption of obedient workers is a recurring theme throughout the crowdshipping literature [14,23,24,25,26,27]. For example, Chen et al. [23] study an offline setting where all delivery requests and shippers are known upfront. Through offline planning, a matching between parcels and drivers is computed. However, such planning algorithms assume a fleet of employees that can be controlled and are hence not directly applicable to crowds of autonomous, self-interested workers.

These publications—if at all—assume only a limited notion of worker autonomy. Often, workers must declare their temporal availability upfront and are assumed to accept any task that lies within the declared availability [25,26]. Sometimes, simple constraints are added, such as vehicle capacity [23,24], a maximal detour length [23,27], or a maximal number of transfers [27].

Up to now, few multi-agent proposals have existed that aim to incorporate task transfers into crowdshipping.

- In Rodríguez-García et al. [28], transfers are performed when two drivers benefit financially, and redundant trips can be avoided. The re-assignments are proposed by a plan manager, which has knowledge about all the delivery tasks, drivers, and their properties.
- In Giret et al. [15] and Rebollo et al. [29], multi-agent systems are combined with complex network-based algorithms to route parcels through the crowd. The proposed system maintains live information about the city’s transportation network and the couriers’ GPS locations.
- In Dötterl et al. [30], we presented an agent-based approach for delivery delay prevention in crowdshipping. When a delay is predicted, a parcel transfer is proposed that both the current deliverer and the prospective new deliverer are allowed to reject.

Besides the challenge of worker autonomy in accepting or rejecting a transfer request, a second issue remains insufficiently addressed: many crowdshipping transfer mechanisms operate on data that is assumed to be available upfront and to remain valid during the entire plan execution. Various proposals perform offline planning, i.e. a plan is precomputed based on data that is assumed to be provided to the algorithm upfront [23,25,26,27]. However, in practice unexpected events can interfere with the plan execution. To react to unexpected events and plan deviations, at least the incorporation of live data is necessary, as proposed by Giret et al. [15] and Rebollo et al. [29]. While transfer methods based on live data already have great advantages over offline methods, they can at most be reactive. Only when the event has already occurred can a reaction be initiated.

To prevent task failures proactively, task outcomes must be predicted. While a task is being executed, the outcome of that task is uncertain—during its execution, it is generally unknown whether the task is going to succeed. However, the decision of whether a task should be transferred strongly depends on the anticipated outcome. To coordinate effective quality-improving task transfers, task outcomes must be accurately predicted.

The combination of crowdshipping with outcome prediction has received little attention so far. One work in this direction is authored by Habault et al. [31], who suggest using machine learning to predict delays in a crowdsourced food delivery platform. However, their proposal strongly differs from our approach: in their proposal, delay prevention is attempted via rerouting—not via task transfers.

In Dötterl et al. [30], we presented an agent-based approach that predicts delivery delays from the workers’ smartphone sensor data. For the delay prediction, the agents use data stream processing and data stream mining. Based on these predictions, the agents negotiate task transfers. This article further extends this work.

We conclude that the literature on task transfers in mobile crowdsourcing to a large degree focuses on planning algorithms. However, offline planning algorithms are unable to react to unexpected events and plan deviations, which regularly occur during plan execution. Existing papers that use task transfers tend to neglect the crowdworkers autonomy and the outcome uncertainty associated with tasks and transfers. In order to effectively address outcome uncertainty, accurate task outcome predictions are a prerequisite.

## 3. Crowdshipping with Collaborative Workers

*Crowdshipping* (sometimes referred to as crowd logistics), as defined in Section 1, is a particularly relevant example of mobile crowdsourcing. In crowdshipping, the delivery of parcels to recipients is outsourced to a crowd of individual workers.

A major goal of the crowdsourcer (usually a logistics company) with regard to the last mile problem is to establish bilateral agreements with crowdworkers regarding some transportation tasks such that an acceptable quality of service is ensured. Among others, this implies that parcels are delivered as soon as possible, and certainly by some deadline. In conventional mobile crowdsourcing, however, it appears that tasks are often abandoned, as crowdworkers usually accept tasks based on their current situation and environmental circumstances. However, this often happens to be a misjudgment, and when this becomes apparent (as the context changes with time), the workers prefer to hand over the task [18].

In the following, we present worker-to-worker parcel transfers as a new approach to improve service quality in crowdshipping. A key element in this respect is the accuracy of the *service quality prediction model* that is used to trigger task transfer decisions. In Section 3.1, different data stream learning methods are applied and analyzed for predicting the delivery success. In Section 3.2, we embed this prediction model into a collaborative agent-based crowdshipping model where task transfer decisions are taken locally and with limited overhead (see also [22]).

### 3.1. Consideration of Outcome Uncertainty by Predicting Delivery Success

Parcel transfers should only be triggered if some delivery task is at risk of failure. Consequently, a key issue is predicting the success of the parcel delivery outcome. Here, we consider that a task has failed when a worker cannot complete it before the deadline. This is only one of several possible key performance indicators for the collaborative case. (for instance one could also take in account the amount of delay). However, in this paper, we aim at comparing collaborative and autonomous crowds, and, especially in the latter case, it seems reasonable to assume that penalties are always applied (on a binary basis) when a task is not successfully accomplished in time. We assume that in both scenarios, all assigned tasks are to be completed necessarily; i.e., delayed tasks cannot just be dropped. This can be assured through specific normative or social control schemes (very high monetary penalties, exclusion from the crowd, loss of reputation, etc.). Notice that in a dynamic environment, the quality of the prediction with regard to this performance indicator depends on the situation awareness of the crowdworkers.

#### 3.1.1. General Prediction Model

The prediction of delivery success can be considered as a binary classification problem. For a crowdworker responsible for a certain parcel, it must be decided whether the parcel can be delivered on time, or in other words, whether the assignment belongs to the class DELAYED or not. More formally, the hypothesis function h() shown in Equation (1) must be learned.

(1) $$\hat{y}=h\left(\vec{x}\right)$$

with the target $\hat{y}\in$ {DELAYED, NOT_DELAYED} and the feature vector $\vec{x}=(x_{1},x_{2},\ldots ,x_{n})$, which contains all available data used to predict the task delivery outcome.

However, it is not sufficient to know only whether an assignment between courier *c* and parcel *p* belongs to class DELAYED; we also need to quantify how certain this prediction is, i.e., the exact value of the delay probability $prob_{delay}\left(c,p\right)$. Fortunately, most machine learning algorithms are able to provide probability estimates of their predictions. For instance, in a decision tree, if a data item has been traversed to a particular leaf node, then the predicted delay probability is the percentage of delayed items out of all items that have been propagated to that leaf node. In scikit-learn, decision_function() and predict_proba()) provides provides such prediction probabilities.

The crowdworker can use three types of data to make a realistic prediction about whether or not a parcel belongs to the DELAYED class:

(i) *Worker capabilities*: each worker has knowledge about her own traveling speed achieved so far, which is given by the current, mean, maximum, and minimum speed.
(ii) *Parcel delivery state*: the current delivery state of a parcel is defined by the remaining distance to the destination and the remaining time until the delivery deadline.
(iii) *Environmental situation*: the traffic situation relevant for parcel delivery can be characterized by the current traffic situation on the remaining delivery route. We assume that each worker agent can obtain information about the traffic density on its route via a central service.

For evaluating the quality of a machine learning approach, we calculate well-known evaluation metrics for classification problems: precision, recall, and F1-score [32]:

- Recall=TP/(TP+FN) is the percentage of the DELAYED parcels that are classified correctly, with true positives (TP), false positives (FP), and false negatives (FN). An optimal recall of 1.0 means that all DELAYED parcels are correctly classified as DELAYED.
- Precision=TP/(TP+FP) is defined by the percentage of the correct classification. The precision is of value 1.0 when all parcels classified as DELAYED are DELAYED.
- The *F1-score* is the harmonic mean of the recall and precision values and provides an aggregated measure of classification quality. A perfect F1-score of 1.0 is given when precision and recall both are 1.0.

#### 3.1.2. Learning from Data Streams

In a crowdshipping system, the prediction of the delivery success must be learned on a stream of continuously arriving data: whenever two crowdworkers, one of whom has a parcel, encounter one another, each of them must estimate the probability with which she could deliver the parcel on time. Classification is the task of predicting the correct label (here: DELAYED or NOT_DELAYED) of a parcel *p* for an unlabeled feature vector $\vec{x_{i}}$ (as introduced in Equation (1). During the training stage, the classification algorithm observes a data stream *D*.

(2) $$D=\{\left(\vec{x_{i}},y^{p}\right)|i=0,1,2,3,\ldots ,m\}$$

where the *i*-th training data item $\left(\vec{x_{i}},y^{p}\right)$ is the feature vector $\vec{x_{i}}$ with the corresponding true target label $y^{p}$. The machine learning algorithm uses this training data to learn a prediction model. This model can be used to predict the still unknown and most probable label ${\hat{y}}^{p}$ for a newly arriving feature vector ${\vec{x}}_{new}$.

Data streams exhibit some characteristic features [33,34]:

- Data arrives continuously online,
- Data streams are potentially infinite and cannot be held completely in memory,
- Data must be processed when they arrive and cannot be retrieved again unless they are permanently stored explicitly,
- Data adapt to temporal changes: concept drift.

When learning on data streams [35], data cannot be clearly separated between training, evaluation, and testing data (as is the case with batch learning). Instead, when predicting whether a parcel will arrive delayed, the correct result is not available until a later time. For each data item, data stream learning goes through a processing cycle ’*predict -> fit model -> evaluate*’ with the following steps:

1. Obtain an unlabeled data item $\vec{x_{i}}$,
2. For $\vec{x_{i}}$: make a prediction ${\hat{y}}^{p}=h\left(\vec{x_{i}}\right)$ for parcel *p* using the current model h(),
3. Determine the true label $y^{p}$ for $\vec{x_{i}}$ (here: when at a later time it is known whether parcel *p* is delayed),
4. Use the new correct pair $\left(\vec{x_{i}},y^{p}\right)$ to train the current model h(),
5. Take the pair $\left({\hat{y}}^{p},y^{p}\right)$ to update statistics for evaluation of the model quality.

Following these steps, we apply an interleaved or *prequential* evaluation approach: Each item is first used to test the model by making a prediction for this previously unseen item. Then, the model is updated (trained) with this item as soon as its label is available.

There are several stream learning algorithms that adapt well-known batch-learning classification algorithms to data streams. Among others, we applied K-nearest neighbors (KNN), random forest, and Hoeffding trees [35,36,37].

#### 3.1.3. Experimental Results for Predicting Delivery Outcome

Using the data streams generated by an agent-based crowdshipping simulator (see Section 3.4), we conducted various experiments for predicting the delivery success. Our machine learning experiments were performed with *River* (https://riverml.xyz/ (accessed on 12 December 2022)) that integrates the data stream learning libraries *scikit-multiflow* (https://scikit-multiflow.github.io (accessed on 12 December 2022)) and créme (https://pypi.org/project/creme/ (accessed on 12 December 2022)). Table 1 shows our results using the data stream versions of KNN, random forest, and Hoeffding.

**Figure 1:**
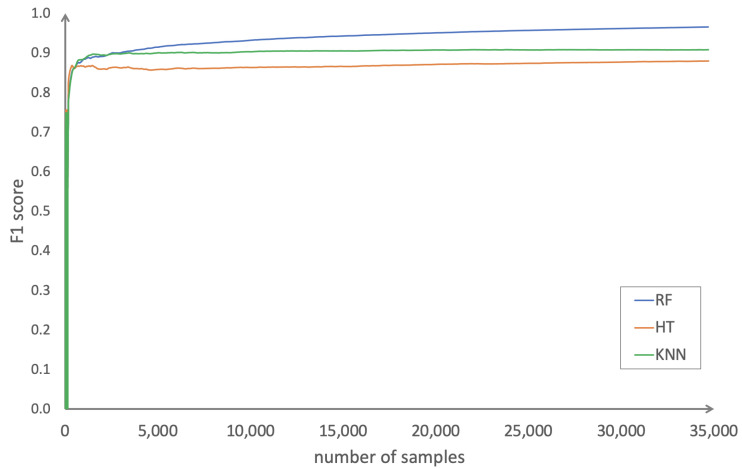
F1-score convergence for random forest (RF), Hoeffding (HT), and KNN.

All applied data stream learning methods behaved almost the same and provided very good prediction results. In particular, ensemble learning with random forest based on n=20 trees yielded precision and recall values of better than 95%. Furthermore, appropriate prediction models were learned fast. Figure 1 shows the convergence of the data stream learning process considering the F1-score for the three machine learning methods. After the arrival of about 2000 data items, the F1-score for all methods reached a value of about 90%. After that, the F1-score was almost constant, only for random forest it was slightly increasing.

When deriving a prediction model, we are interested not only in its accuracy but also in understanding how the predictions are inferred. A feature importance value can be viewed as a percentage expressing how much a particular feature contributes to the prediction. Feature importance helps us understand which features are mainly used in the prediction and may allow us to reduce the feature space, making the model training much faster.

Table 2 shows how the importance of the features introduced in Section 3.1.1 is distributed among the different types.

Our data stream learning approach is based on many different features. To provide a better overview, we aggregated the feature importance values of the individual features according to three categories by summing up the individual values. The most important features relate to the delivery state of the parcel with an overall importance of about (54%). In particular, the remaining delivery time is by far the most important feature (36%). The features associated with the workers’ capabilities, specifically how fast they are, have an overall importance of 19%. The environmental features sum up to about 27% and describe the remaining path with the corresponding traffic states.

### 3.2. A Collaborative Agent Model for Task Transfers in Crowdshipping

Task transfers are particularly challenging in the crowdshipping domain, as either crowdworkers must physically meet at some place at a specific time or the parcel needs to be stored in a secure place and picked up later by the newly responsible worker. In traditional fleet management systems such parcel transfers can be arranged in a top-down manner by the logistics company, but the decentralized nature of the crowdshipping approach calls for methods that rely on peer-to-peer interaction [30].

In the following, we propose an agent-based collaboration model towards the crowdshipping problem (see also [22]). In this model, the option of performing a transfer of parcel *p* from its bearer *i* to another worker *j* is explored only when the couriers *i* and *j* “encounter”, i.e., when their locations are close to each other. In this case, the expected quality of service provided by *i* performing *p* is compared to *j*’s service quality executing *p*, and if the latter exceeds the former, the parcel *p* is transferred from *i* to *j*.

Service quality here does not need to be defined exclusively in terms of potential task delay but may also consider factors such as fuel cost, CO${}_{2}$ footprint, reliability, etc.

The model avoids a major source of complexity: parcel transfers can be effectively implemented on-the-fly because, as mentioned above, both workers are already near to one another. Of course, such a stance necessarily implies less emphasis on strategic issues.

Algorithm 1 summarizes this collaborative worker agent model for parcel transfers. It is obvious that it depends on two key elements:

1. A function for predicting the *service quality* of a worker with respect to a delivery task is needed. The service quality function $F_{qual}\left(c,p\right)$ specifies how well a crowdworker *c* is able to achieve the delivery of a parcel *p*; i.e., a high value means better quality.
2. The concept of *vicinity* needs to be specified so as to be able to identify the set $CS_{c}^{t}$ of candidate couriers. Notice that the more restrictive the notion of vicinity is, the more seldomly couriers encounter one another, and the fewer task transfer opportunities exist.

For instance, the service quality can be specified by the prediction of the delivery outcome learned by the prediction model introduced in Section 3.1. Thus, the service quality function in line 2 can be defined as:

(3) $$F_{qual}\left(c,p\right)=1-prob_{delay}\left(c,p\right)$$

A parcel should be transferred to a worker of higher quality, i.e., with a lower delay probability.

The notion of vicinity is used in Line 5 of the algorithm in order to determine the set of possible candidate workers. Several different options exist to specify the vicinity of two workers. A simple and practical model for vicinity is the definition of a cell-based environment model, where a grid is placed over the urban operation area.

Whenever a courier worker with an assigned parcel moves (in discrete time steps in Lines 4–10), it looks for a more suitable worker in the vicinity. According to Algorithm 1, Line 5, nearby located crowdworker agents are potential candidates for a parcel transfer. By definition, all agents who are currently in the same grid cell in a time step as the courier worker are in vicinity to each other and, therefore, are considered as candidates. The prediction model learned in Section 3.1 is now used to estimate the delivery success probability of each candidate. A parcel transfer takes place if at least one of the candidate workers has a smaller delay probability as the courier worker (Lines 7–8). The parcel is transferred to that candidate with smallest delay probability. For the sake of simplicity, it is assumed that candidates do not yet have an assigned parcel, and transfers do not take any time (duration = 0).
**Algorithm 1：** Collaborative parcel transfer algorithm.  1:**procedure**Collaborative ParcelTransfer  2:    Define service quality function $F_{qual}\left(c,p\right)$;  3:    **for each** time step *t* **do**  4:        **for each** courier *c* with parcel *p* **do**  5:             Determine the candidate set $CS_{c}^{t}$,      ▹ all couriers $c_{i}$ in vicinity to *c*  6:             $cand_{best}=\left\{c^{*}\in CS_{c}^{t}|F_{qual}\left(c^{*},p\right)=\max_{\forall c_{i}\in CS_{c}^{t}}\left(F_{qual}\left(c_{i},p\right)\right)\right\}$  7:             **if** $F_{qual}$(c,p) < $F_{qual}$($cand_{best},p$) **then**     ▹candidateisbettersuited  8:               Transfer parcel *p* from *c* to $cand_{best}$  9:             **end if**10:        **end for**11:    **end for**12:**end procedure**

### 3.3. Experimental Evaluation of Collaborative Agent Approach

Using the agent-based simulator introduced in Section 3.4 and the prediction models of Section 3.1, we conducted extensive experiments of our proposed collaborative crowdshipping approach.

As the baseline of experiments, we implemented a conventional crowdshipping approach, where every worker has to deliver its initially assigned parcel without any possibility of exchange. In a simulation run comprising 600 parcels, the conventional approach without any parcel transfers results in 39% of the parcels being delayed, i.e., 234 of 600 parcels cannot be delivered in time. The mean completion time over all parcels is 16.28 min.

Table 3 shows the simulation results of our collaborative crowdshipping model with the different prediction models presented in Section 3.1. It can be seen that the percentage of delayed parcels can almost be reduced by half (from 39% to approx. 21%), and the mean completion time drops by 25 % from around 16 min to 12 min. The fourth column lists the total number of transfer activities and the fifth column the number of reassigned parcels. It can been seen that some parcels have been exchanged more than once. For instance, 249 transfers from a current courier to a candidate worker took place, and 210 parcels were affected. The last column lists the number of parcels in time or delayed after being transferred, respectively. For instance, taken the 210 reassigned parcels, 162 could have been delivered in time after the transfer, whereas 48 parcels were still delayed.

The three prediction models applied provide similar results in terms of delay probabilities and completion times. This was to be expected because the results of the prediction models in Section 3.1 also hardly differ from each other.

Overall, the results demonstrate that collaborative crowdshipping with delivery outcome prediction has the potential to significantly improve service quality of last mile delivery processes.

### 3.4. Agent-Based Crowdshipping Simulator

In order to evaluate the feasibility of our task transfer models, we developed an agent-based crowdshipping simulator (an extended version of the simulator presented in [38]). The simulator implements a simplified model of the environment, in particular how crowdworkers behave and how the traffic situations change. The simulator can be operated with different task transfer coordination strategies that realize different levels of agent autonomy. The implementation of a concrete strategy can be plugged into the simulator.

To make the simulation as realistic as possible, we employed real-world GPS data to simulate the physical movements of the agents. We chose open data of a bicycle sharing system because of its conceptual similarity to a crowdshipping system. In both domains, there are users who register in the system, physically move around the urban area, and log out of the system. Specifically, we employed the GPS data from the Bike Sharing System of Madrid (BiciMAD) (https://opendata.emtmadrid.es/Datos-estaticos/Datos-generales-(1) (accessed on 12 December 2022)). The data set includes the rides logged by users and the GPS events recorded during each ride: start timestamp, start location, end location, and GPS traces. Based on BiciMAD data we simulated parcel delivery in the urban city center of Madrid. The appearance of a worker agent, its start location and destination, and its route were derived from the BiciMAD data set.

**Figure 2:**
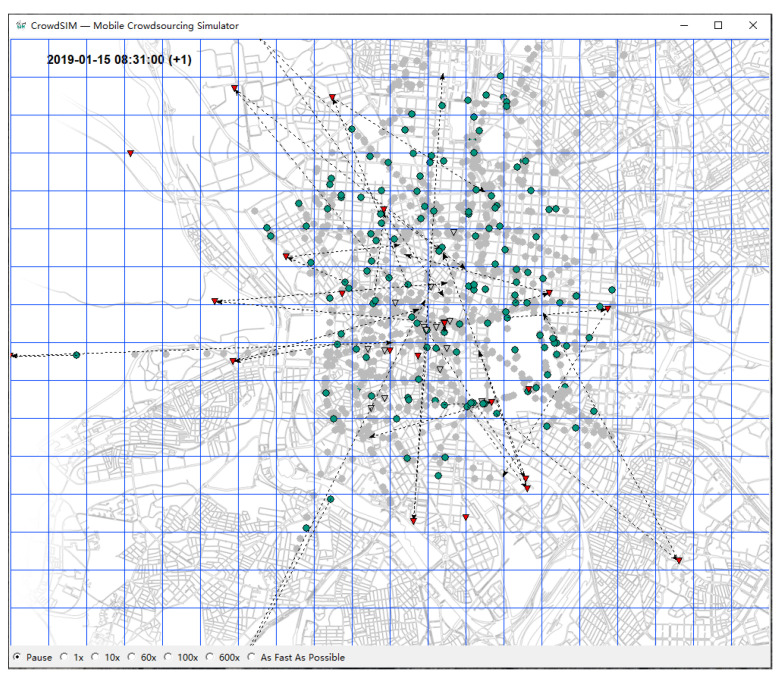
Agent-based simulator at work.

The simulation runs were conducted with several thousand worker agents and several hundred parcels to be delivered. The parcels had randomly selected origin and destination locations within the operating area. Figure 2 shows a screenshot of a simulation run. (Red triangles visualize remaining, gray triangles already visited tasks. Green circles show current, gray circles past agent positions).

#### 3.4.1. Environmental Model

Crowdshipping usually operates in highly populated urban areas. In an urban area, the speed at which a crowdworker moves is strongly determined by the current traffic situation. In order to realize a simple traffic model, we divided our operating area into a grid structure. We assumed that each grid cell can be in one of the following traffic states: *normal traffic, slow traffic, and traffic jam*. For crowdworkers moving on the road, the state of the cells affects their velocity. For instance, in a cell with state *traffic jam,* the agents using the road do not move at all (travel with 0 km/h). In a cell which is in the state *normal traffic*, all agents travel with their individual preferred speeds, which is determined by the class to which an agent belongs (see Section 3.4.2).

Of course, the traffic situation in an urban area changes very dynamically, and as a consequence, the velocity of road users also varies over time. We used a time-discrete Markov chain to model the changing traffic state in a rather simple way. In the simulation, the cell states are updated frequently.

#### 3.4.2. Crowdworker Agents

The crowd consists of individual worker agents who are registered in the system and interested in delivering parcels. The model distinguishes three types of crowdworker agents with different properties: *walk, bike, and motorbike*. Each agent is defined by an arrival time, start location, end location, and a route with several predefined stopover points. This means, the agents move on different paths of different lengths.

The walk agents and the bike agents do not move on roads; therefore, their speed does not depend on the traffic situation. These two agent types move with a constant speed: walk with 2 m/s and bike with 5 m/s. The motorbikes travel on the road, and their speed consequently depends on the traffic situation of the grid cell where they are currently located.

For a more detailed description of the simulator and the concrete parameters used for experimentation, please refer to the crowdshipping version [22] and crowdsensing version [2].

## 4. Crowdsensing with Autonomous Workers

Crowdsensing systems, as defined in Section 1, rely on a crowd of volunteer individuals collecting sensor data at specific locations. Crowdsensing inherits many advantages from crowdsourcing, such as access to a scalable workforce. It is known that crowdsensing has many advantages over sensing with fixed sensor installations [5].

In this section, we show that task transfers can be used effectively to instill and maintain coordination in a workforce of *autonomous workers*. For this purpose, we apply the *Auction-based Task Transfers* (ATT) approach introduced in [2] to the crowdsensing domain. Crowdworkers are perceived as mobile agents capable of performing sensing tasks at their current location (e.g., using their mobile phones). Still, different from the setting analyzed in Section 3, here we assume that crowdworkers are *individually rational* and only accept an additional task when the reward obtained from its successful completion outweighs the corresponding cost. We show that by allowing such self-interested crowdworkers to transfer tasks among each other on-the-fly, the overall performance of the system can be improved without significantly compromising their autonomy.

It should be noted that as in the crowdsensing domain, all tasks are fully virtual; they can be easily transferred between workers without the need for handovers. No parcel or other item has to be handed over between the workers, so agents do not have to visit a same location. Therefore, as opposed to the crowdshipping domain discussed in Section 3, such task transfers can in principle be performed regardless of the positions of the involved agents. This implies that once agreed upon by the involved crowdworkers, task transfers are *immediate* and thus cannot fail due to changing circumstances.

### 4.1. Autonomous Crowdsensing with Task Transfers

In this subsection, we describe our market-based coordination mechanism with self-interested crowdworkers. Therefore, the concepts of task rewards, penalties, and expected task outcome are introduced, so as to define the notion of expected task utility. Subsequently, the protocol for task transfer auctions among crowdsensing agents is described. We finally outline rational agent strategies for triggering auctions and bidding.

#### 4.1.1. Expected Utility of Task Sets

In the crowdsensing domain, each crowdworker $a_{i}$ has a utility function $U_{i,t}\left(S\right)$, which indicates $a_{i}$’s utility for being assigned a set of sensing tasks *S*. Each sensing task $\theta_{j}\in S$ generates a reward $r_{j}$ for the agent completing it successfully and a penalty $p_{j}$ if the crowdworker does not execute the corresponding service with the required quality. Task utilities are computed as follows:

(4) $$\begin{array}{cc} U_{i,t}\left(S\right) & =-C_{i,t}\left(S\right)+Rev\left(S\right)\\ \; & =-C_{i,t}\left(S\right)+\sum_{\theta_{j}\in S}O_{j}r_{j}-\sum_{\theta_{j}\in S}\left(1-O_{j}\right)p_{j} \end{array}$$

where $O_{j}$ denotes the binary task outcome (successful execution or failure).

This utility function expresses what the crowdworker incurs in costs for performing the tasks in *S*, which are compensated by the revenue that the worker obtains by performing them. The worker’s revenue can be negative in cases where the worker fails to successfully complete some of their tasks and has to pay task penalties. The revenue Rev(S) is computed by summation of all rewards and subtraction of all penalties. The worker-specific cost function $C_{i,t}\left(S\right)$ is subadditive and increases weakly monotonically in the size of *S*. It models not only the worker’s monetary expenses such as gasoline, vehicle maintenance, and vehicle insurance, etc., but also includes the compensation that the worker expects for the intangible factors, such as the required effort and lost time for performing the tasks.

The task set utility $U_{i,t}\left(S\right)$ can be computed once the outcomes $O_{j}$ of all tasks $\theta_{j}\in S$ are known. For decision making, a worker $a_{i}$ needs access to the expected set task utility, which is given as:

(5) $$\begin{array}{cc} EU_{i,t}\left(S\right) & =-E\left[C_{i,t}\left(S\right)\right]+E\left[Rev\left(S\right)\right]\\ \; & =-E\left[C_{i,t}\left(S\right)\right]+\sum_{\theta_{j}\in S}E\left[O_{j}\right]r_{j}-\sum_{\theta_{j}\in S}\left(1-E\left[O_{j}\right]\right)p_{j} \end{array}$$

To determine the expected task set utility in practice, online data stream learning techniques can be used to compute the expected task outcomes $E[O_{j}]$. For this, we rely on techniques similar to the ones described in Section 3.1.

#### 4.1.2. Task Transfer Auctions

**Figure 3:**
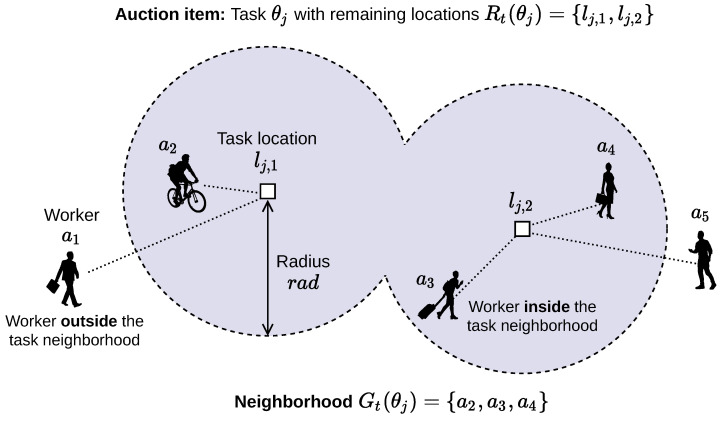
Task neighborhood example.

Crowdsensing agents are registered to a software platform that sets up a computational market where sensing tasks can be traded among the agents. If an agent $a_{i}$ believes that it is beneficial for it to give away one of its sensing tasks $\theta_{j}$, it can trigger an online auction. For this, it announces $\theta_{j}$ as the “good” to be auctioned to the agents in its neighborhood $G_{t}\left(\theta_{j}\right)$. Figure 3 depicts different neighborhoods of task $\theta_{j}$ depending on the position $l_{j}{,}_{k}$ of the agent that it is currently assigned to.

An agent interested in becoming the new assignee of task $\theta_{j}$ may then respond with a bid, indicating the amount *b* that it is willing to pay for this. Notice that the reward $r_{j}$ or penalty $p_{j}$ are attributed to the agent that finally completes the task $\theta_{j}$, so there is an incentive to “pay” for receiving an additional task $\theta_{j}$. The auction rules determine the auction winner $a_{k}$, who will become the new assignee of task $\theta_{j}$, as well as the transfer reward $r_{z}$ that it has to pay to $a_{i}$. In this work, we use a one-shot second-price (Vickrey) auction for this purpose as it makes strategic bidding more difficult [39,40].

#### 4.1.3. Agent Strategies

A crowdsensing agent is continuously monitoring its set *S* of tasks $\theta_{j}$ assigned to it. If, based on the expected task outcome $E[O_{j}]$, the expected utility of giving away $\theta_{j}$ plus a potential transfer reward $r_{z}$ exceeds the expected utility of its current set of sensing tasks *S* (Δeu>0), it will trigger an auction. Still, the transfer reward $r_{z}$ is unknown at the time of launching an auction, and in our implementation, agents are conservative and only launch an auction when Δeu>0, even with $r_{z}=0$.

Similarly, an agent will join an auction as a bidder if, based on the expected task outcome $E[O_{j}]$, the expected utility of adding $\theta_{j}$ to its set of sensing tasks *S* exceeds the expected utility of *S* alone. Let *b* denote the difference between both values, which constitutes its true valuation of “winning” $\theta_{j}$ in the auction. As we are using Vickrey auctions, there is no need for the agents to employ a complicated bidding strategy because, assuming the agents’ valuations are independent, the dominant strategy for all agents is to bid their true valuations. Therefore, we assume our rational agent’s bid value in the online auction just to be *b*.

### 4.2. Evaluation

In this section, we explore how well the ATT approach performs in different crowdsensing scenarios. We start by outlining the experimental setup to then present results on delay percentages as well as average and individual profits.

#### 4.2.1. Experimental Setup

For our experiments, we used an instantiation of the simulator described in Section 3.4 and enriched the BiciMAD data set with synthetic data to simulate the arrival of new tasks over time. Each task consisted of a set of locations from the crowdsensing operating area. For our experiments, we chose a 1500 m radius in the center of Madrid, which covered the urban city center where many trips of the BiciMAD data set (and therefore the simulated agents) pass through. Each task consisted of three locations, which were aligned to form a chain such that each successor location was 500 m apart from its predecessor. All tasks have deadlines, rewards, and penalties: deadlines were set to 30 min after task occurrence. In our experiments, we assumed that task failures are undesirable but acceptable with a certain ratio. As a scenario that seems reasonable, we set the reward and the penalty to the same amount (EUR 5). In [2], the effect of different reward–penalty combinations was investigated. As the crowdsensing operating area was rather small, we set the task neighbourhood radius to be infinite, i.e. task transfer auctions were broadcast to all agents.

We compare our approach *ATT* with different baselines:

- *Not* (No transfers): Using this strategy, the crowdworkers do not transfer tasks between each other. Even when tasks are at risk of failing, crowdworkers cannot offer the task to other workers but have to try to finish the tasks themselves.
- *Random* (Random transfers): Using this strategy, for each time step and task, there is a random chance that the task is transferred to the nearest worker without any tasks. This baseline models a “blind” transfer behavior: the transfers are initiated without predictions and the task recipient is chosen without predictions.
- *Forced* (Forced transfers): Using this strategy, the system identifies the most promising task transfers and executes them without the workers’ consent. The system makes predictions about the task outcome, and if a better worker can be identified, for which the task success has a higher probability, the task is transferred to that worker. This baseline is useful to evaluate how the system would perform if the system objective could be maximized if workers were not autonomous.

To gain a deeper insight into how *ATT* and the baselines behave in different settings, we compared them in three scenarios:

- *Scenario 1* (Base scenario): In this scenario, we generated 50 tasks per hour (600 total) and set the incident probability to 5%.
- *Scenario 2* (Increased system load): In this scenario, we generated 100 tasks per hour (1200 total) and set the incident probability to 5%. The purpose of this scenario was to explore how the different strategies respond to an increase in tasks.
- *Scenario 3* (Increased environment hostility): In this scenario, we generated 50 tasks per hour (600 total) and set the incident probability to 10%. The purpose of this scenario was to explore how the different strategies respond to higher disruption levels.

For each scenario–strategy pair, we executed 30 simulation runs and report the mean of delay percentage and worker profits.

#### 4.2.2. Delay Percentage

**Figure 4:**
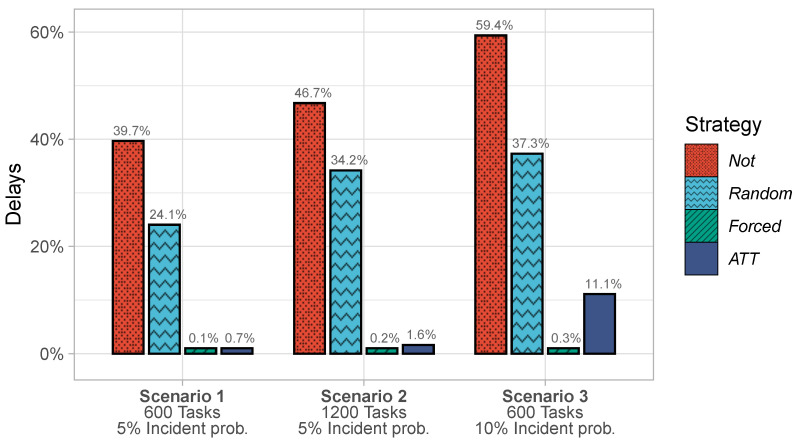
Delays under four strategies in three crowdsensing scenarios.

The bar chart in Figure 4 shows the delay percentages for the four strategies in the three scenarios.

Across the three scenarios, *Forced* and *ATT* performed significantly better than *Not* and *Random*. Both *Not* and *Random* struggled in all scenarios and were affected by higher task counts and incident probabilities. *ATT* could handle the increase in tasks but started to struggle when the incident probability was high. *Forced* produced hardly any delays in any of the three scenarios.

The poor performance of *Not* and *Random* can be explained by the high incident probability. The excellent performance of *Forced* and *ATT* shows that task transfers help to effectively reduce delays. The superiority of *Forced* and *ATT* compared to *Random* shows that those transfers must be guided by accurate task outcome predictions; transfers are only effective if they are based on predictions rather than randomness.

*Forced* produced fewer delays than *ATT*, which is unsurprising. While the effectivity of *ATT* was restricted by the workers’ autonomy, *Forced* can initiate arbitrary task transfers that it considers useful for delay prevention. In other words, the reason for the superior performance of the *Forced* strategy is its larger action space: *Forced* can transfer tasks to any worker, while *ATT* can transfer tasks only to willing workers.

#### 4.2.3. Average Profits

**Figure 5:**
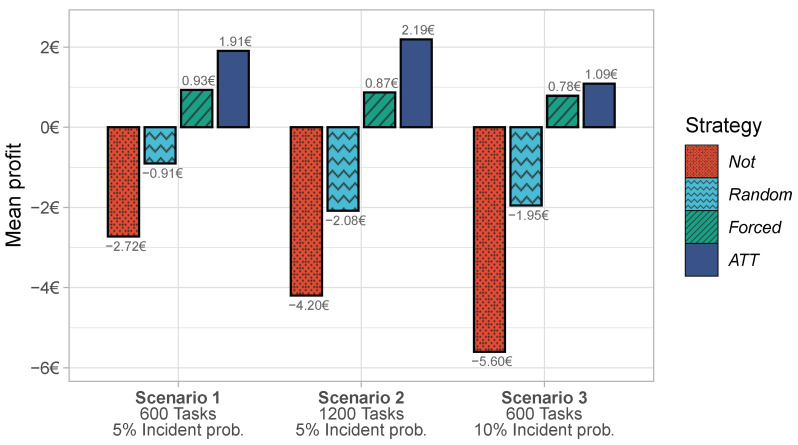
The observed profits under four strategies in three crowdsensing scenarios.

We now investigate how *ATT* compares with the baselines in terms of worker profits. The mean profits of the participants using the four strategies in the three different scenarios are shown in Figure 5. Across the three scenarios, *Not* performed worst and yielded high negative profits. The *Random* strategy also performed too poorly to achieve positive worker profits. On average, both *Forced* and *ATT* provided positive profits to workers with *ATT* clearly dominating *Forced*.

The negative profits generated by *Not* and *Random* are explainable by the high number of delays that are observed under these strategies. The delays result in penalty payments, which cause the negative profits. *Forced* and *ATT* resulted in very few delays and hence lead to many reward payments and fewer penalties. The plot reveals that the negative profits take more extreme values than the positive profits. This is because the rewards and penalties paid to and charged from the workers are further reduced by the costs. For instance, under *Not* the workers not only suffer penalties but incur additional costs as well.

*ATT* leads to higher profits than *Forced*: While *Forced* generates the fewest delays, on average, workers gain more using *ATT*. The reason is that *Forced* minimizes delays, ignoring the costs incurred by the workers in the process. To prevent a delay, *Forced* can force a worker to take a large detour and incur high costs. It may also take a task from a worker; that worker loses the opportunity to obtain the reward but is not compensated for the costs that the worker already incurred. By contrast, *ATT* only transfers tasks to workers for whom the promised reward is higher than the costs for completing the task.

#### 4.2.4. Individual Profits

We have seen that both *ATT* and *Forced* result in positive profits—on average. While the mean profit is a useful indicator, it conveys little information about the fate of individual workers. Even in scenarios where the mean profit is high, there may exist a notable number of workers with negative profits. To gain a deeper insight into the profits of individual workers, we analyze the profits under *ATT* and *Forced* in more detail.

**Figure 6:**
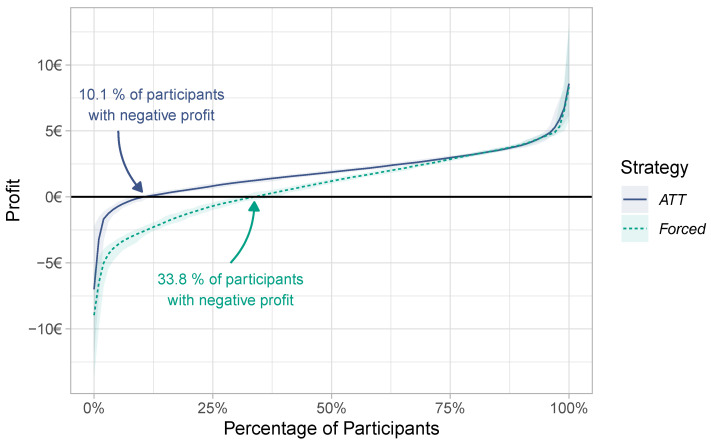
Profit distribution under the *ATT* and *Forced* strategy in Scenario 1.

The cumulative frequency graph in Figure 6 shows how the profits in Scenario 1 under *ATT* and *Forced* are distributed over the individual participants. Under the *Forced* strategy, 33.8% of participants obtained a profit of EUR 0 or less; i.e., a large fraction of participants would suffer under the forced task transfers. In contrast, using *ATT*, only 10.1% of participants obtained a profit of EUR 0 or less.

Most workers with negative profits under *ATT* are individuals who have invested costs to perform tasks but were not able to finish them and collect the reward. In the absence of task failures (in this experiment, only 0.7% delays occurred), travel costs are the only source that diminishes a participant’s profit. Hence, participants with negative profits were not able to fully recover their travel costs, but *ATT* helped them to avoid penalty payments for failed tasks. From the worker perspective, *ATT* is clearly preferable to *Not* and *Forced*.

## 5. Conclusions

Mobile crowdsourcing has proven to be a versatile problem-solving paradigm that can bring value to many domains where spatial tasks have to be performed quickly and sustainably at low costs. These tasks are performed by individual crowdworkers in dynamic and uncertain environments. However, unexpected events, such as traffic issues or competing obligations, can disrupt tasks executions, resulting in unreliable system behavior and low service quality. In this paper, we argue that supporting peer-to-peer task transfers to better-suited crowdworkers is a promising approach to prevent task failures. In particular, we identified two major problems in coordinating crowdworkers in dynamic environments and discussed corresponding solutions:

- First, crowdworkers must be situation-aware; that is, they must identify emerging issues early enough to trigger task transfers. We proposed a data stream learning approach to predict the probability that a task assigned to a particular crowdworker will be successful. Experimental results showed that various data stream learning methods can be used to predict service quality with high accuracy. This enables crowdworkers to make a realistic and continuous assessment of whether a task transfer is useful.
- Second, task transfers between crowdworkers must be coordinated. We proposed two different approaches to task coordination: an opportunistic approach to crowdshipping with collaborative, but non-autonomous workers, and a market-based model with autonomous workers for crowdsensing. Both approaches showed that the service quality could be significantly improved by task transfers.

In general, through the coordinated interaction of crowdworkers, many task failures can be prevented, resulting in a more reliable system with higher stakeholder satisfaction.

From a managerial perspective, the applicability of our approach depends on the characteristics of the crowd system and the underlying business model. In a decentralized crowd of individually rational agents, it is important that the coordination mechanism respects the autonomy of the workers. Instead, in a system with top-down management, where agents have agreed to accept orders (e.g., as employees), the overall quality of the system can be further optimized. However, some workers may suffer from a decrease in individual profit. In both cases, we conclude that a good mechanism for predicting task success is essential. From a micro-level perspective, crowdworkers would usually be interested in maintaining their autonomy. They would prefer the market-based approach presented, which represents a good compromise between workers’ and system interests.

From a micro-level perspective, crowdworkers would usually be interested in maintaining their autonomy. They would prefer the market-based approach presented, which represents a good compromise between worker and system interests.

In future work, we will focus on some aspects of the practical applicability of our approach. Our assumption that task transfers take no time is a simplification of reality (crowdworkers need to stop, hand over the transfer, etc.). We will explore how different notions of transfer costs will affect the task transfer chains. In this context, we will explore different ways of splitting rewards (penalties) for (un)successful delivery tasks between the participating crowdworkers so that the transfers required by our mechanism are also individually rational for all stakeholders. This includes investigating the manipulability of information in general and of the prediction models in particular. We will also look into more homogeneous experimental scenarios to further improve the significance of the quantitative comparison of the approaches.

Furthermore, the possibility to negotiate handover locations at runtime may offer advantages. For example, the two workers could meet at a public location that is close to both of their planned routes. In this case, the detours could be smaller and handovers faster, resulting in faster and more sustainable plans.

Our market-based approach uses Vickrey auctions [39] for agreements on a single transfer and a single transfer attribute. Using a more sophisticated auction, we could expand our protocol to multidimensional auctions. Instead of only determining the price by the auction, multi-dimensional auctions could be used to negotiate further transfer attributes, such as the transfer deadline and handover location at runtime. Moreover, combinatorial auctions may bring further benefits. Using combinatorial auctions, workers could give away and obtain bundles of tasks, which in some cases can be more attractive than the individual tasks.

## Figures and Tables

**Table 1:** Experimental results for the prediction of the delivery success.

Prediction Model	Precision	Recall	F1-Score
Random Forest (*n* = 20)	0.957	0.974	0.966
KNN (*K* = 10)	0.912	0.904	0.908
Hoeffding	0.879	0.881	0.880

**Table 2:** Aggregated feature importance.

Feature	Importance
Worker capability (current, max, min, mean speed)	0.19
Parcel delivery state (remaining time, remaining distance)	0.54
Environmental situation (traffic states and distances on remaining path)	0.27

**Table 3:** Experimental results of collaborative parcel transfers.

Prediction Model	Delay	Completion Time	Transfers	Reassigned Tasks	In Time/ Delayed
Random Forest (*n* = 20)	20%	12.55 min	249	210	162/48
KNN (*K* = 10)	22%	12.67 min	256	216	169/47
Hoeffding	21%	11.97 min	227	196	159/37
Convent. Crowdshipping	39%	16.28 min	–	–	–

## Data Availability

The source code used to generate the simulation data study is available from the authors upon request.

## Abbreviations

The following abbreviations are used in this manuscript:

ATT	Auction-based task transfer
FN	False negative
FP	False positive
HT	Hoeffding Tree
KNN	K-nearest neighbors
RF	Random Forrest
TP	True positive
